# Comprehensive Proteome and Acetylome Analysis of Needle Senescence in *Larix gmelinii*

**DOI:** 10.3390/ijms25136824

**Published:** 2024-06-21

**Authors:** Xuting Zhang, Jinyuan Shan, Jiaxiu Wang, Yanxia Zhang, Feiyun Yang, Bin Liu, Lifeng Zhang, Guojing Li, Ruigang Wang

**Affiliations:** 1 Key Laboratory of Plants Adversity Adaptation and Genetic Improvement in Cold and Arid Regions of Inner Mongolia, Inner Mongolia Agricultural University, Hohhot 010018, China; 2 College of Food Science and Engineering, Inner Mongolia Agricultural University, Hohhot 010018, China; 3 State Key Laboratory of Tree Genetics and Breeding, Research Institute of Forestry, Chinese Academy of Forestry, Beijing 100091, China

**Keywords:** *Larix gmelinii*, needle senescence, proteome, acetylome

## Abstract

Leaf senescence is essential for the growth and development of deciduous trees in the next season. *Larix gmelinii*, a deciduous coniferous tree, exhibits its most distinctive feature by turning yellow in the autumn and eventually shedding its leaves, resulting in significant changes in its appearance during the fall. Lysine acetylation plays an important role in diverse cellular processes; however, limited knowledge is available regarding acetylations in the needle senescence of *L. gmelinii.* In this study, the proteomics and acetylated modification omics of two phenotypic leaves, yellow and green (senescent and non-senescent) needles, were analyzed before autumn defoliation. In total, 5022 proteins and 4469 unique acetylation sites in 2414 lysine acylated proteins were identified, and this resulted in the discovery of 1335 differentially expressed proteins (DEPs) and 605 differentially expressed acetylated proteins (DAPs) in yellow versus green needles. There are significant differences between the proteome and acetylome; only 269 proteins were found to be DEP and DAP, of which 136 proteins were consistently expressed in both the DEP and DAP, 91 proteins were upregulated, and 45 proteins were down-regulated. The DEPs participate in the metabolism of starch and sucrose, while the DAPs are involved in glycolysis and the tricarboxylic acid cycle. Among them, DEPs underwent significant changes in glycolysis and citric acid cycling. Most of the enzymes involved in glycolysis and the citrate cycle were acetylated. DAPs were down-regulated in glycolysis and up-regulated in the citrate cycle. In all, the results of this study reveal the important role of lysine acetylation in the senescence of *L. gmelinii* needles and provide a new perspective for understanding the molecular mechanism of leaf senescence and tree seasonal growth.

## 1. Introduction

*Larix gmelinii*, an excellent stress-resistant coniferous tree species, is widely distributed and has important economic and ecological value [1]. It is a deciduous tree among coniferous, with obvious seasonal changes, and can be used as an ideal model for studying needle senescence. However, up to date only a few studies have involved the proteomics of larch. For example, Zhang and collaborators used proteomics to study the mechanism of drought stress in *Larix olgensis* families [2]; Han and collaborators analyzed the formation of adventitious root development in hybrid larch with proteome [3]; and proteomics were widely used in the study of somatic embryogenesis in larch [4,5,6,7]. No studies to date have conducted a comprehensive analysis of the proteomic in needle senescence in *L. gmelinii*.

Post-translational modifications (PTMs) are complex processes that modulate proteins covalently by introducing new functional groups and modifying or removing the original functional groups; these modifications occur frequently after the proteins have been fully translated [8,9]. Lysine acetylation (LysAc) is a ubiquitous, reversible and highly conserved PTMs of both histones and non-histone proteins of prokaryotes and eukaryotes, and affects protein functions through diverse mechanisms, including by regulating protein stability, enzymatic activity, subcellular localization and crosstalk with other post-translational modifications, and by controlling protein-protein and protein-DNA interactions [10]. LysAc plays a key role in plant physiological and metabolic processes, including the cell cycle, flowering time, responses to environmental conditions, such as light or pathogen attack, root and shoot development, hormone signaling, and epigenetic processes [11,12].

Early LysAc investigations focused mainly on histones [13,14]. Subsequently, with the advantages of anti-acetyllysine-based enrichment and high-resolution mass spectrometry (MS), acetylome studies have been begun to be performed in plants, and the first systematic studies of LysAc demonstrated that during *Arabidopsis thaliana* development, LysAc modification participates in the regulation of central metabolic enzymes [15,16]. To date, plant Lys-acetylproteome analyses have focused mainly on *A. thaliana* [17], *Vitis vinifera* [18,19], *Pisum sativum* [20], *Glycine max* [21], *Medicago truncatula* [22], *Capsicum annuum* [23], *Oryza sativa* [24,25,26], *Triticum aestivum* [27], *Zea mays* [28], *Camellia sinensis* [29,30], *Populus tremula* [31]. However, there are currently few research reports on LysAc in coniferous trees and the understanding of this modification in conifers is limited. LysAc analysis was carried out only in desiccated somatic embryos of *Picea asperata* [32].

In order to better understand the mechanism of needle senescence in *L. gmelinii*, we present here for the first time comprehensive proteome and acetylome profiling of two different colored needles before defoliation in the autumn. Our findings provide important information on the molecular basis of needle senescence, which helps to enrich the LysAc information of conifers and enrich the mechanism of leaf senescence in woody plants.

## 2. Results

### 2.1. Changes in Physiological Indicators of L. gmelinii Needle Senescence

**Figure 1:**
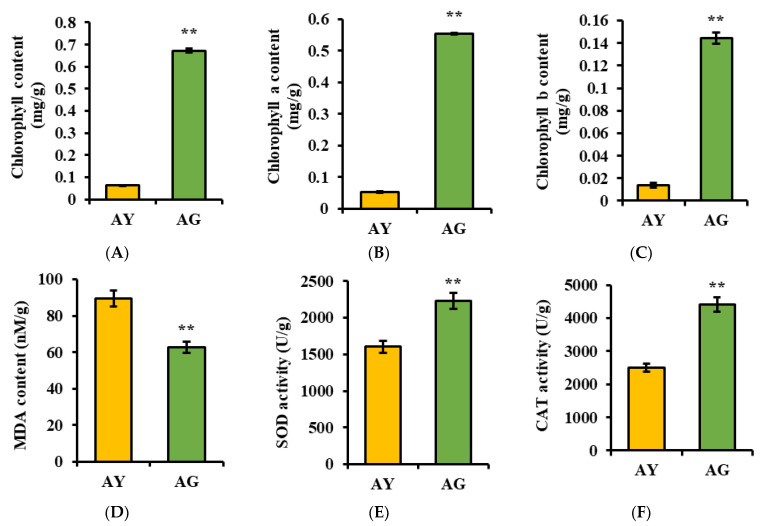
Changes in the physiological indices of spicule senescence in *L. gmelinii* included chlorophyll (**A**), chlorophyll a (**B**), chlorophyll b (**C**), MDA content (**D**), SOD (**E**), and CAT activities (**F**). AY and AG are senescing (yellow) and non-senescing (green) needles, respectively. (**, *p* < 0.01, Student’s *t*-test).

The most obvious feature of leaf senescence is the degradation of chlorophyll, accompanied by the yellowing and eventual abscission of the leaves [33]. Our results showed the content of chlorophyll a and chlorophyll b, and the total chlorophyll in the yellow needles was significantly lower than that of the green needles. The increase in MDA content in the yellow needles indicates a high degree of lipid peroxidation in the plant cells. The activity of SOD and CAT showed a decreasing trend in yellow needles, indicating a weakened ability of yellow needles to eliminate the reactive oxygen species produced during plant metabolism (Figure 1). The above results indicate that the different color of needles before defoliation are due to different periods of senescence.

### 2.2. Identification of Proteins in Needle Senescence of L. gmelinii via Proteomics and DEPs Annotation

In order to study the mechanism of needle senescence, different colored needles before defoliation were selected for proteomic analysis. Quality control validation of MS data, including the mass error, peptide ion score, peptide molecular weight, peptide count, and protein sequence coverage distributions, revealed that the results of our analysis were accurate and reliable (Figure S2A–D). The proteomic analysis identified 28,498 (94.32%) unique peptides from a total of 30,213 detected peptides (a false discovery rate, FDR ≤ 0.01). In total, 5022 protein groups (FDR ≤ 0.01) were identified based on these peptides, among which 3082 protein groups were quantified. Three-dimensional PCA showed good repeatability and significant differences between groups, PCA1, PCA2, and PCA3, which could explain 40.9%, 15.1%, and 11.2% of the total variance, respectively (Figure S2E). Based on a *p* value ≤ 0.05 level, the cutoff value of 1.5-fold for up-regulation or down-regulation was used to define the effect of needles senescence on proteins. Using these criteria, a total of 1335 DEPs were identified in AY vs. AG, including 757 and 578 DEPs with up-regulated and down-regulated (Figure S2F, Table S1).

Gene ontology databases were used to categorize all of the quantified DEPs. In terms of the number of DEPs, cellular process, and cell and catalytic activity were predominant in biological processes, cellular components, and molecular function, respectively (Figure S3A). To reveal the subcellular localization of the DEPs in the needle senescence, the prediction indicated that the majority of the validated acetylated proteins were distributed in diverse subcellular locations with 45% in chloroplast, and a number of acetylated proteins were also localized to the cytoplasm (24%), nucleus (13%), and mitochondria (5%) (Figure S3B).

**Figure 2:**
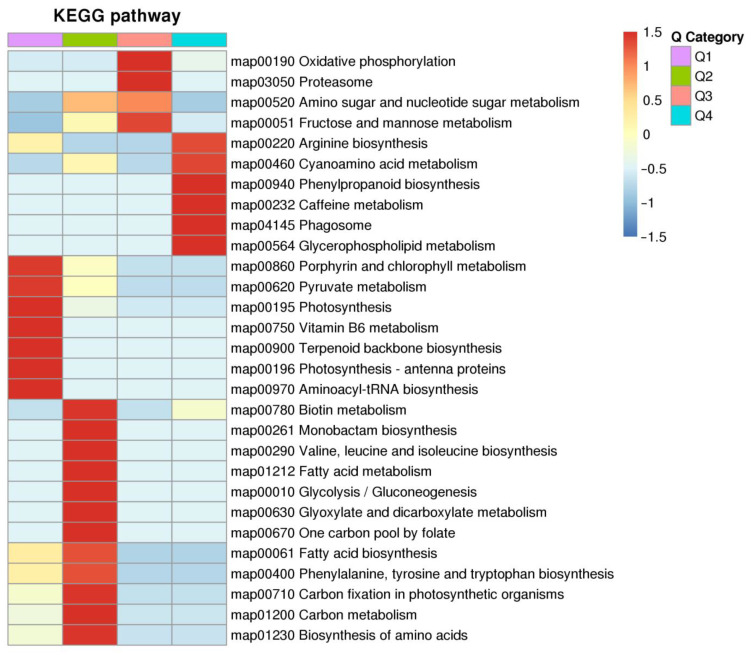
KEGG pathway enrichment analysis of DEPs, red indicates abundant and blue indicates least abundant.

To elucidate the functional differences of these proteins, the DEPs were analyzed for KEGG enrichment based on clustering analysis. According to their level of differential expression, the DEPs were divided into four parts, Q1 to Q4, Q1 DEPs are down-regulated with a ratio AY vs. AG inferior to 0.5-fold (355 DEPs); Q2 DEPs are down-regulated with a ratio AY vs. AG between 0.5- and 0.667-fold (223 DEPs); Q3 DEPs are up-regulated with a ratio AY vs. AG between 1.5- and 2-fold; Q3 (328 DEPs and Q4 DEPs) and Q4 are up-regulated with a ratio AY vs. AG higher than 2-fold (429 DEPs). There are significant differences in the enrichment of DEPs in different metabolic pathways in Q1, Q2, Q3, and Q4. The up-regulated DEPs in Q3 and Q4 participate in oxidative phosphorylation, fructose and mannose metabolism, arginine biosynthesis, phenylpropane biosynthesis, and other metabolic pathways. The down-regulated DEPs in Q1 and Q2 participate in porphyrin and chlorophyll metabolism, photosynthesis, terpenoid backbone biosynthesis, fatty acid metabolism, carbon metabolism, and other metabolic pathways (Figure 2).

### 2.3. Lysine-Acetylation of Needle Senescence in L. gmelinii

**Figure 3:**
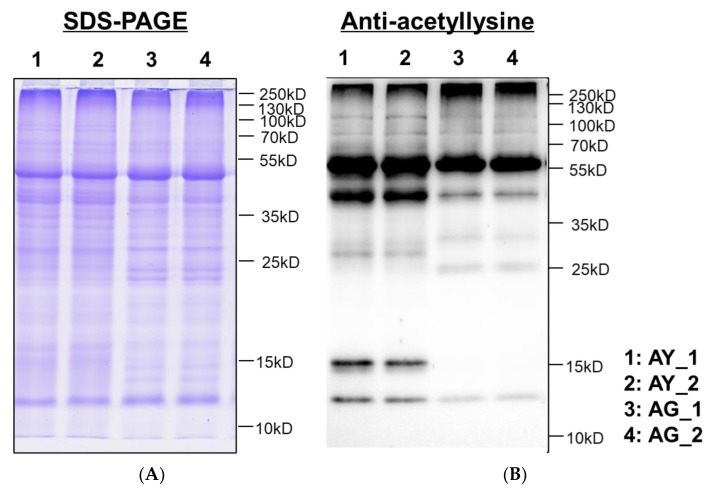
Western blotting analysis of proteins using a pan anti-acetyllysine antibody in needle senescence of *L. gmelinii*. Image of SDS-PAGE stained with coomassie blue (**A**), Western blotting of the needles proteins with pan anti-acetyllysine (**B**). AY and AG are senescing and non-senescing needles at test site A, respectively. 1 and 2 represent two replicates of AY; 3 and 4 represent two replicates of AG.

To acquire a general view of LysAc in needle senescence of *L. gmelinii* with the green and yellow needles, multiple major proteins with the molecular weights similar to histones and non-histones were successfully identified by western blotting using an anti-acetyllysine antibody (Figure 3), showing that the level of LysAc was higher in the yellow needles (AY). For example, the signals of 10–55 KD bands were enhanced upon anti-acetyllsine antibody detection in the yellow needles (AY) compared with the green needles (AG). Studies on other modifications have found that 2-hydroxybutyrylation, malonylation, lactylation, and ubiquitination exhibit differences in modification in needle senescence, but there is little difference between succinylation and crotonylation (Figure S4). On this basis, to obtain global insight into the large-scale dataset of LysAc sites in needle senescence of *L. gmelinii*, we used antibody-based affinity enrichment and high-resolution MS to identify and analyze the acetylated proteins in needles with the distinct color yellow and green of *L. gmelinii*.

**Figure 4:**
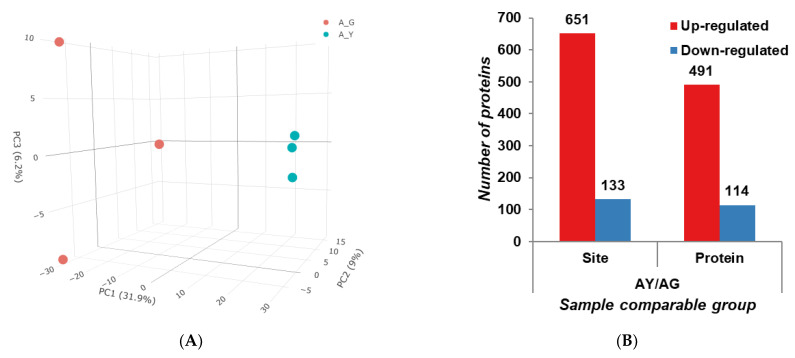
Overview of acetylome analysis in the needle senescence of *L. gmelinii*. Three-dimensional PCA analysis of temporal acetylome data (**A**); the PC1, PC2, and PC3 axes represent the loading of each protein quantification value in the first, second, and third principal components, respectively. Numbers of up- and down-regulated DAPs in AY vs. AG (**B**).

In total, we successfully identified 4469 unique acetylation sites in 2414 acetylated proteins in the needle senescence of *L. gmelinii*; the average degree of acetylation was 1.85 sites per protein, and the number of LysAc sites in each protein ranged from 1 to 8. Here, our data extend the inventory of lysine-acetylated proteins in plants, providing information to reveal the role of LysAc in needle senescence as well as development. According to the analysis of the three-dimensional PCA results, it was found that AY and AG are clearly distinguished and are ideal materials for studying needle senescence (Figure 4A). Based on a *p* value ≤ 0.05 level, the cutoff value of 1.5-fold for up-regulated or down-regulated was used to define the effect of needles’ senescence on modified proteins and modified sites. Using these criteria, a total of 605 DAPs with 784 modified sites were identified in AY vs. AG, including 491 proteins with 651 sites that were up-regulated and 114 proteins with 133 sites that were down-regulated (Figure 4B, Table S2). Consistent with the western blotting results, LysAc showed significant changes in premature senescent needles.

### 2.4. Analysis of LysAc Motifs in Needle Senescence of L. gmelinii

**Figure 5:**
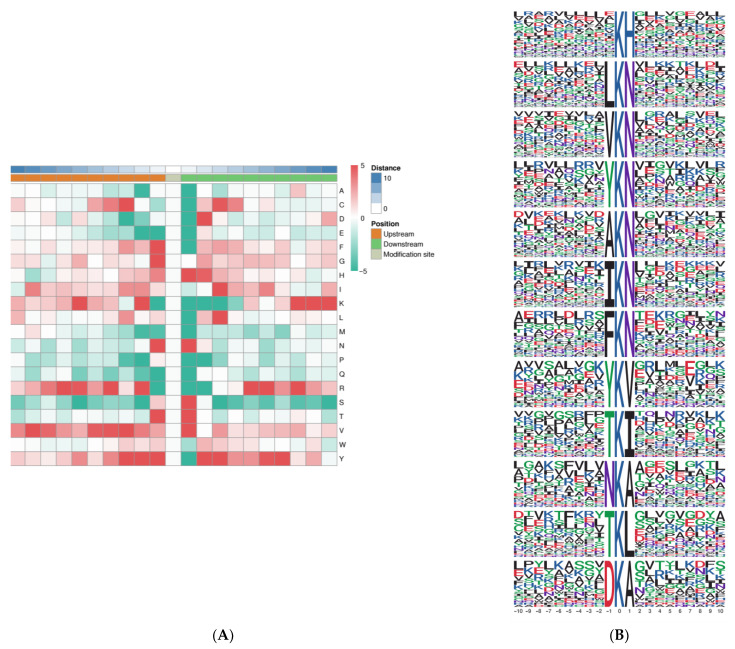
Sequence motifs returned by motif-x (motif length = 21, ten amino acids upstream and downstream of the acetylation site). Heat map showing the over-representation of amino acid residues at positions from −10 to +10 from the acetylated lysine residue relative to the overall proteome background distribution. Red indicates abundant and green indicates least abundant (**A**). 20 common amino acids: A, (Alanine); C, (Cysteine); D, (Aspartic acid); E, (Glutamic acid); F, (Phenylalanine); G, (Glycine); H, (Histidine); I, (Isoleucine); K, (Lysine); L, (Leucine); M, (Methionine); N, (Asparagine); P, (Proline); Q, (Glutamine); R, (Arginine); S, (Serine); T, (Threonine); V, (Valine); W, (Tryptophan); Y, (Tyrosine). Motif-x analysis of over-expressed motifs around the acetyl site of identified senescent needles of *L. gmelinii* (**B**). Sequence logos for acetylation sites detected in proteins include KH, LKN, VKN, YKN, AKN, IKN, FKN, YKV, TKI, TKL, NKA, and DKA.

In addition, to elucidate the properties of Kac and to identify specific amino acids adjacent to Kac sites, we analyzed the amino-acid sequences flanking Kac sites using iceLogo (Figure 5A). Overall, five amino-acid residues were overrepresented in the +1 position: histidine (H), asparagine (N), serine (S), threonine (T), and valine (V), and five amino-acid residues were overrepresented in the −1 position: phenylalanine (F), glycine (G), asparagine (N), threonine (T), and tyrosine (Y). Valine (V) and tyrosine (Y) were significantly enriched in proximal positions (from −6 to +6) surrounding the Kac site. In more distant positions, lysine (K) and valine (V) residues were most considerably enriched on both sides of the Kac site.

The preferred amino acid residues surrounding the LysAc sites have been identified in plants. To identify the possible specific motifs flanking acetylated lysine, we analyzed the overrepresented motifs of the amino acids using the motif-x algorithm. 32 significantly enriched motifs were identified (2.0 < fold increase < 13.2); the top 12 over-represented motifs are depicted in Figure 5B (fold increase > 8.0). Inspection of these 12 motifs demonstrated that two types of amino acid residues surround the acetylated sites: the enrichment of N or A on the +1 position, and mostly N is in the +1 position; the enrichment of Y or T on the −1 position. These data revealed the conserved motif model and residue preferences for acetylation in the needle senescence of *L. gmelinii* and thus provide valuable information for the acetyl site prediction of unknown acetyl proteins.

### 2.5. Functional Characterization of Lysine-Acetylated Proteins in Needle Senescence of L. gmelinii

**Figure 6:**
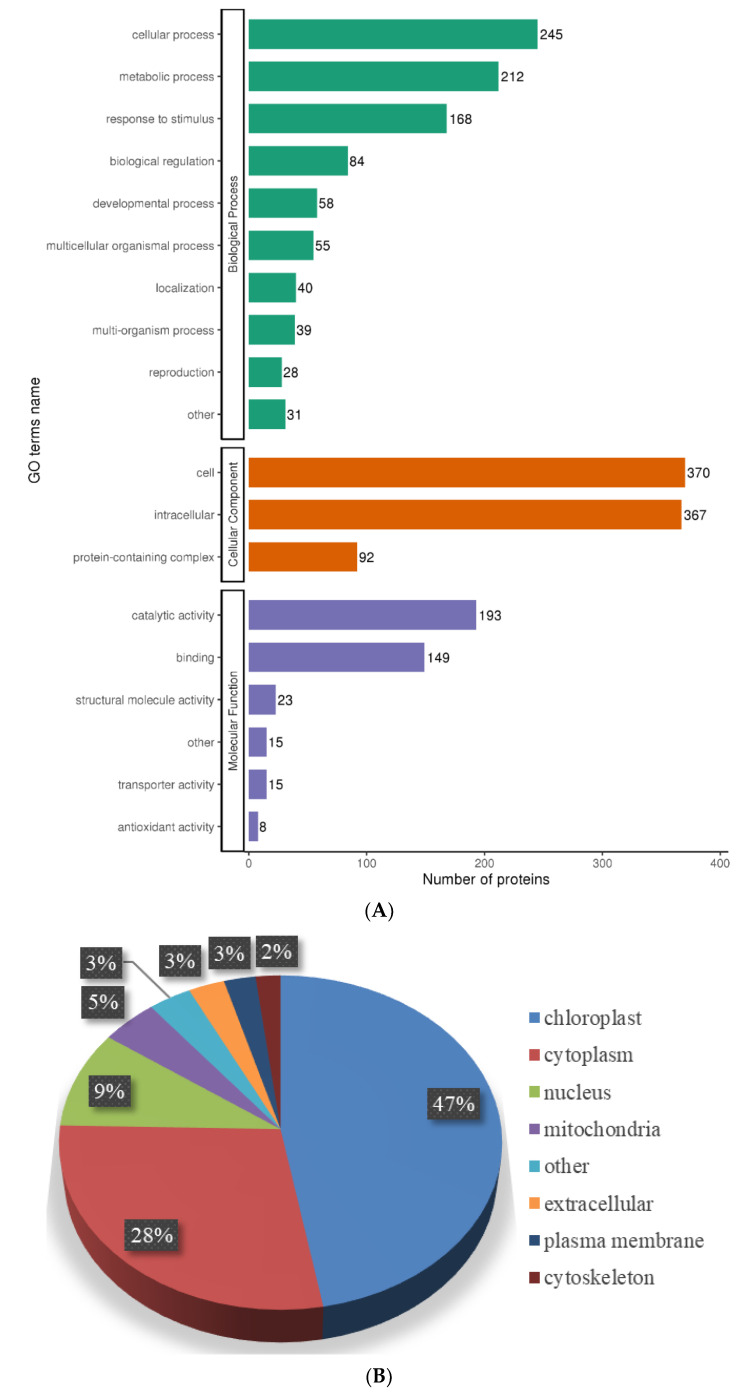

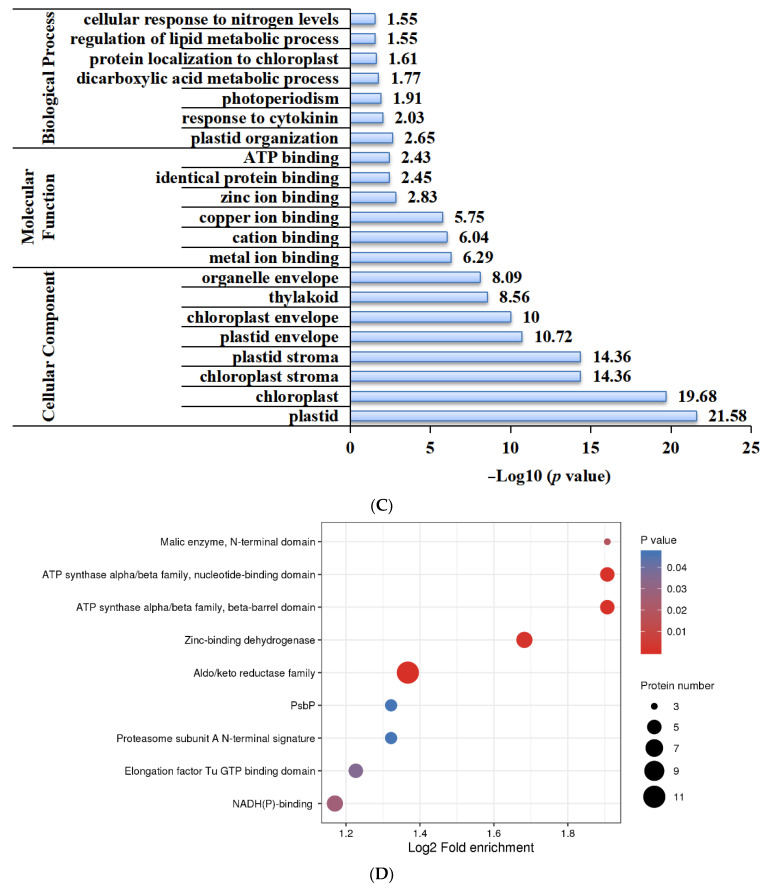

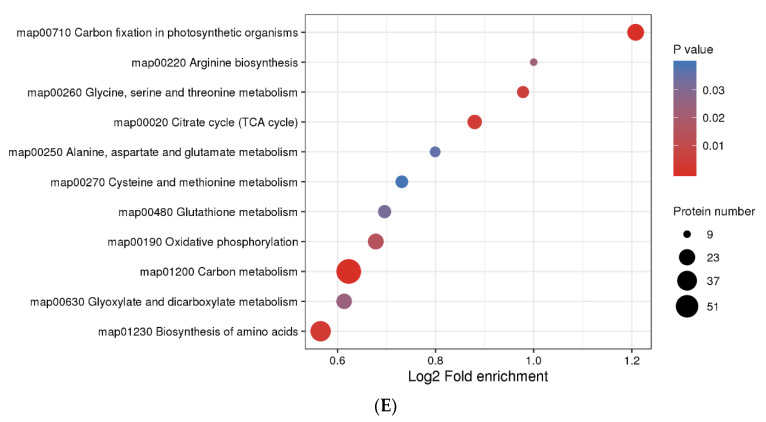
Functional classification of DEPs. Gene ontology (GO) analyses of proteins by biological process, cellular component, molecular function (**A**); Subcellular location (**B**); GO enrichment analysis (**C**); Protein domain enrichment analysis (**D**); KEGG pathway enrichment analysis (**E**).

To better understand the function of the lysine acetylome in the needle senescence, the lysine-acetylated proteins were subjected to GO classification. In the functional classification of biological processes, the acetylated proteins of the premature senescence needles were involved in cellular processes, metabolic processes, and the response to stimulus; for the cellular component ontology, the acetylated proteins were mainly classified into the cell and intracellular. In the molecular functions, the analysis demonstrated that the acetylated proteins were associated with catalytic activity and binding (Figure 6A).

To reveal the subcellular localization of acetylation in the needle senescence, we conducted subcellular location prediction (Figure 6B). The prediction indicated that the majority of the validated acetylated proteins were distributed in diverse subcellular locations with 47% in chloroplasts, suggesting that LysAc in this compartment plays an important role in needle senescence. Furthermore, a number of acetylated proteins were also localized to the cytoplasm (28%), nucleus (9%), and mitochondria (5%).

Furthermore, we carried out GO enrichment analysis to determine which functional terms were targeted for LysAc in the needle senescence (Figure 6C). In the analysis of cellular components, chloroplast and plastid complexes were significantly enriched. Accordingly, the structural constituents of the chloroplast were significantly enriched in the molecular function category. For the molecular function, the DAPs were significantly enriched in metal ion binding, identical protein binding, and ATP binding. Our data also demonstrated a significant enrichment of DAPs in various biological processes, including the response to cytokinin, photoperiodism, protein localization to chloroplast, and the regulation of lipid metabolic process, indicating the pivotal role of LysAc in virtually all fundamental metabolic processes.

To search for enriched protein domains in the acetylated proteins of *L. gmelinii*, we applied protein domain enrichment analysis to the acetylome data, the reductase family, ATP synthase, and NAD(P)-binding domain which were significantly enriched in the DAPs (Figure 6D).

The DAPs were mapped to KEGG pathways to better understand their general functions in needle senescence. The results showed that amino acid metabolism-related pathways, such as the biosynthesis of amino acids, glutathione metabolism, cysteine and methionine metabolism, alanine, aspartate and glutamate metabolism, glycine, serine and threonine metabolism, arginine biosynthesis; and carbon metabolism, oxidative phosphorylation, and the citrate cycle were highly represented by the DAPs in the AY vs. AG (Figure 6E).

### 2.6. Correlation between Proteome and Acetylome in Needle Senescence of L. gmelinii

Most of the DAPs (51.96%) displayed differences only in the LysAc but not in the protein level. 1066 DEPs (79.85%) are only differentially expressed at the protein level, while only 269 proteins were found to be DEP and DAP, of which 136 proteins were consistently expressed in both the DEP and DAP, 91 were up-regulated and 45 were down-regulated. In summary, there are significant differences between the proteome and acetylome, suggesting the importance of the post-translational LysAc of these proteins.

**Figure 7:**
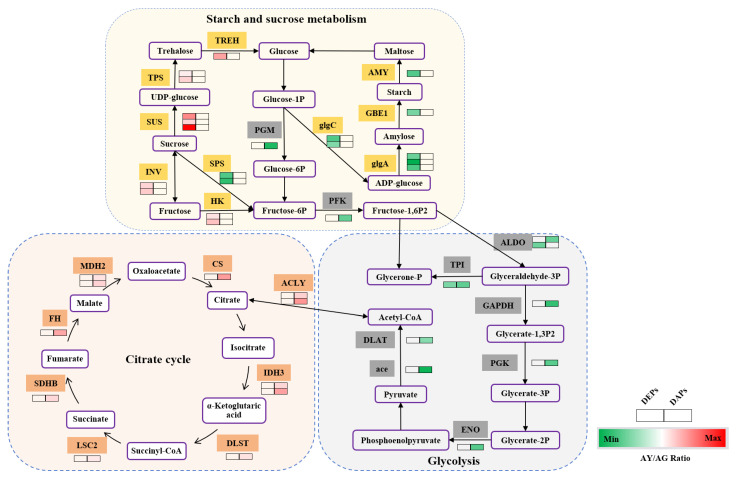
Overview of the DEPs and DAPs that are involved in primary metabolic processes in the needle senescence of *L. gmelinii*. The orange part indicates the citrate cycle, yellow indicates starch and sucrose metabolism, and gray indicates glycolysis. The primary metabolic processes include starch and sucrose metabolism, glycolysis, and the citrate cycle pathways. The colored scale bar depicts fold change: red indicates up-regulated and green indicates down-regulated. SUS, sucrose synthase; TPS, trehalose phosphate synthase; TREH, trehalase; INV, invertase; HK, hexokinase; glgC, glucose-1-phosphate adenylyltransferase; glgA, starch synthase; GBE1, 1,4-alpha-glucan branching enzyme; AMY, amylase; SPS, sucrose-phosphate synthase; PGM, phosphoglucomutase; PFK, 6-phosphofructokinase; ALDO, fructose-bisphosphate aldolase; TPI, triosephosphate isomerase; GAPDH, glyceraldehyde 3-phosphate dehydrogenase; PGK, phosphoglycerate kinase; ENO, enolase; ace, pyruvate dehydrogenase E1 component; DLAT, pyruvate dehydrogenase E2 component; ACLY, ATP citrate (pro-S)-lyase; CS, citrate synthase; IDH3, isocitrate dehydrogenase; MDH2, malate dehydrogenase; FH, fumarate hydratase; SDHB, succinate dehydrogenase; LSC2, succinyl-CoA synthetase beta subunit; DLST, ihydrolipoamide succinyltransferase. Protein information is listed in Table S3.

Notably, there is a significant difference in protein and lysine acetylation levels (Figure 7), as well as significant differences in DEPs’ expression involved in starch and sucrose metabolism pathways. DEPs such as SUS, TPS, TREH, INV, and HK were up-regulated; glgC, glgA, GBE1 and AMY were down-regulated. There is a significant difference in glycolysis and the citrate cycle at the level of lysine acetylation, with DAPs such as PGM, PFK, ALDO, TPI, GADPH, PGK, ENO, ace, and DLAT in the glycolysis being down-regulated, but DAPs such as ACLY, CS, IDH3, MDH2, FH, SDHB, LSC2, and DLST in the citrate cycle being up-regulated.

## 3. Discussion

This study analyzed the proteome and acetylome of needle senescence in *L. gmelinii*. In total, 5022 proteins and 2414 lysine acylated proteins were identified, and this resulted in the discovery of 1335 DEPs and 605 DAPs. Only 269 proteins were found to be DEP and DAP, of which 136 proteins were consistently expressed in both the DEP and DAP. Therefore, there are significant differences between translation and PTM in regulating needle senescence.

Leaf senescence in autumn leads to leaf color change [34]. The most obvious feature of leaf senescence is the degradation of chlorophyll, accompanied by the yellowing and eventual abscission of the leaves [35]. Our results showed the content of chlorophyll a, chlorophyll b, and the total chlorophyll in the yellow needles was significantly lower than in the green needles (Figure 1). Similarly, in the KEGG enrichment analysis of DEPs, it was found that DEPs in the chlorophyll metabolism and photosynthesis were significantly downregulated in the yellow needles (Figure 2). These results indicate that the different color of the needles before defoliation are due to a different period of senescence, which is an ideal material for studying needle senescence in *L. gmelinii*.

The role of sugars in senescence has been widely discussed in recent years, and the sugars have been reported as growth and photosynthetic rate regulators [36]. The photosynthetic rate decreased together with the sugar levels in a mature leaf [37]. In *Arabidopsis* and tobacco, the sugar metabolites decreased gradually before the onset of leaf senescence [38,39]. Similarly, it was found that carbohydrate levels decreased during the senescence of sunflowers [40]. Moreover, carbohydrate biosynthesis-related genes were upregulated in leaves during the growth to maturation stage but downregulated in the maturation to senescence stage [41,42]. However, there is limited research on carbohydrate metabolism in leaf senescence at the translation level. In this study, it was found that DEPs are significantly involved in carbohydrate metabolism processes, especially in starch and sucrose metabolism (Figure 7), and the enzymes involved in sucrose degraded branches were up-regulated, while SPS involved in sucrose synthesis were down-regulated and the enzymes involved in starch synthesis were down-regulated. In summary, at the translation level, carbohydrate metabolism plays an important role in regulating needle senescence.

Recently, with the development of quantitative acetylproteome, a large number of non-histone proteins with lysine acetylation modification have been discovered, and the universality of its existence in biology and the importance of its function have also been highlighted [11]. Non-histone protein lysine acetylation modification is abundant in different tissues, organs, and organelles of plants [16,43]. It is widely involved in various metabolic processes during the growth and development, the acetylated proteins are mainly involved in carbon metabolism (Glycolysis/Gluconeogenesis, TCA cycle and pentose phosphate pathway) and stress response. For example, a drying treatment can improve the germination ability of *P. asperata* somatic embryos, and acetylated proteins are mainly involved in carbon metabolism and lipid metabolism [32]. Meng and collaborators studied the proteome of rice seeds at different development stages, and found that almost all of the enzymes in glycolysis and TCA cycle were acetylated at full bloom [44]. This study also found that most enzymes in glycolysis and the TCA cycle were acetylated of the needle senescence in *L. gmelinii* (Figure 7); consistent with reports in other plants, almost all of the enzymes involved in glycolysis and the TCA cycle undergo LysAc [11]. The autumn leaf senescence of woody plants is accompanied by dormancy, in preparation for adapting to the winter environment [45]. LysAc affects carbohydrate metabolism and switches of carbon and energy flux, further regulating dormant buds. In the dormant buds of hybrid poplar, there was a slight decrease in LysAc during dormancy release [31]. This study suggests that up-regulated LysAc was likely to be involved in the inactivation of the TCA cycle to divert carbon flux from energy production to dormancy and storage deposits in needle senescence. In conclusion, acetylation modification plays an important role in needle senescence, and this study also enriched acetylation modification in *L. gmelinii*.

## 4. Materials and Methods

### 4.1. Plant Materials

The needles of *L. gmelinii* begin to turn yellow in autumn in early October, but some of them still remain green and turn yellow relatively late. The needles with distinct color of the same variety and the same age before they are shed were collected from Wuchuan county (41°1′36 N, 111°48′25 E) of Hohhot City, Inner Mongolia Autonomous Region. The Figure S1 shows the growth of *L. gmelinii* at the time of collection (10 October). There are significant differences in the color of the needles among different individuals of *L. gmelinii*, representative senescing (yellow, AY) and non-senescing (green, AG) needles, the collected needles were snap-frozen with liquid nitrogen for subsequent research.

### 4.2. Determination of Physiological Indexes Indicators

Chlorophyll was extracted from different *L. gmelinii* samples using extract solution (ethanol and acetone volume ratio 1:1) and analyzed according to Lichtenthaler [46]. The same samples were used for malondialdehyde (MDA), catalase (CAT), and superoxide dismutase (SOD) activity analysis, according to the manufacturer’s instructions (Shanghai Youxuan Biotechnology Co., Ltd., Shanghai, China). This data were averaged from three replicates.

### 4.3. Protein Extraction

*L. gmelinii* needles were added with liquid nitrogen and ground to powder. 4 times the volume of phenol extraction buffer (containing 10 mM dithiothreitol, 1% protease inhibitor, 3 μM TSA, 50 mM NAM) was added and then the mixture was decomposed by ultrasonic wave. An equal volume of Tris balanced phenol was added. After centrifuging at 5500× *g* for 10 min at 4 °C, 5 times volume of 0.1 M ammonium acetate/methanol was added to the supernatant to precipitate overnight, and the protein precipitation was washed with methanol and acetone respectively. Finally, the precipitate was redissolved with 8 M urea, and the protein concentration was determined by BCA kit (Beyotime, Shanghai, China).

### 4.4. Western Blotting

The presence of LysAc and other modifications in needles (AY, AG) with different colors was demonstrated by western blotting. Briefly, the protein sample was diluted with SDS loading buffer, and 20 µg of protein from each sample was separated via 12% SDS-PAGE and electro-blotted onto a polyvinylidene fluoride membranes. The blot was then probed with the primary antibody, anti-acetyllysine antibody (PTM-101; Lot: 10167J809; 1:1000 dilution), anti-succinyllysine antibody (PTM-419; Lot: 105032317G009; 1:1000 dilution), anti-crotonyllysine antibody (PTM-502; Lot:1037267K306; 1:1000 dilution), anti-2-hydroxyisobutyryllysine antibody (PTM-802; Lot: 13592312JB09; 1:2000 dilution), anti-malonyllysine antibody (PTM-902; Lot: 23056103K312; 1:1000 dilution), anti-ubiqutin antibody (PTM-1107; Lot: 21671155JA18; 1:2000 dilution), and the anti-lactyllysine antibody (PTM-1401RM; Lot: K082701; 1:1000 dilution) (PTM Bio, Hangzhou, China), followed by incubation with a horseradish peroxidase-conjugated secondary antibody (Thermo, Norristown, PA, USA) at a 1:10,000 dilution.

### 4.5. Protein Trypsin Digestion

The same amount of protein for enzymolysis was taken and the final concentration of 20% TCA was added. Vortex mixing at 4 °C precipitation was carried out for 2 h. After centrifuging at 4500× *g* for 10 min, the supernatant was discarded and the precipitate was washed with precooled acetone 2–3 times. After the precipitation was dried in the air, the final concentration of 200 mM TEAB was added, the precipitation was dispersed by ultrasound, and trypsin was added at the ratio of 1:50 (protease:protein, M/M) for enzymolysis overnight. Dithiothreitol (DTT) was added to a final concentration of 5 mM, and reduced at 56 °C for 30 min. Then, iodoacetamide (IAA) was added to make the final concentration 11 mM, and incubated at room temperature for 15 min.

### 4.6. Lysine-Acetylated Peptide Enrichment

To enrich lysine-acetylated peptides, the peptide was dissolved in IP buffer solution (100 mM NaCl, 1 mM EDTA, 50 mM Tris-HCl, 0.5% NP-40, pH 8.0). The supernatant was transferred to the acetylated resin (PTM Bio, Hangzhou, China) that has been washed in advance, performed at 4 °C overnight with gentle shaking. After the incubation, the resin was washed 4 times with IP buffer solution, twice with deionized water, and finally with 0.1% trifluoroacetic acid eluent. Elution was performed three times to elute the resin-bound peptides, and the eluate was collected and vacuum freeze-dried.

### 4.7. Proteomic Analysis by LC–MS/MS

The peptides and acetylated peptides were dissolved in the mobile phase A of liquid chromatography and then separated using the NanoElute ultra-high performance liquid system (TiMessageTOF, Bruke). Mobile phase A is an aqueous solution containing 0.1% formic acid and 2% acetonitrile; mobile phase B is a solution containing 0.1% formic acid and 100% acetonitrile. The peptides were separated by the ultra-high performance liquid system and then injected into the Capillary ion source for ionization and then into the timsTOF Pro (Thermo) mass spectrometer for analysis. The ion source voltage was set to 1.6 kV, and the peptide precursor ions and their secondary fragments were detected and analyzed using high-resolution TOF. The scanning range of the secondary mass spectrum was set to 100–1700. The data acquisition mode used the Parallel Accumulation Serial Fragmentation (PASEF) mode. After a first-level mass spectrum was collected, PASEF mode was performed 10 times to collect the second-level spectra with the charge number of precursor ions in the range of 0–5. The dynamic exclusion time of the tandem mass spectrometry scan was set to 30 s to avoid repeated scans of the precursor ions.

### 4.8. Database Search and Date Analysis

The MS/MS data obtained were processed using Maxquant (v1.6.15.0) search engine, and the search database is *Larix gmelinii*_123599_TX_20201209.fasta (43,993 sequences) transcriptome. The anti-database was added to calculate the false positive rate (FDR) caused by random matches. The minimum length of the peptides was set to 7 amino acid residues, the maximum number of peptide modifications was set to 5, and FDR was set to 1% for the accuracy of identification at the three levels of spectrum, peptide, and protein; at least one specific peptide must be included to identify the protein. The identified proteins were annotated with common functional databases, including GO, KEGG, InterPro, COG, and STRING database, and the adjusted *p* value < 0.05 and Fold Change ≥ 1.5 as were selected as the standard, the differential expression proteins (DEPs), LysAc proteins (DAPs), and sites were screened.

### 4.9. Motif Analysis

The model of the sequences comprising the ten amino acids upstream and downstream surrounding the LysAc sites in all the acetylated proteins was analyzed using soft motif-x. The differences were screened according to the quantitative results; all the database protein sequences were used as a background database parameter. The minimum number of occurrences was set to 20. Emulate original motif-x was ticked and other parameters with default. The characteristic sequence would be considered to be a motif of the modified peptide.

## 5. Conclusions

This study analyzed the proteome and acetylome of needle senescence in *L. gmelinii* for the first time. In total, 5022 proteins and 2414 lysine acylated proteins were identified, and this resulted in the discovery of 1335 DEPs and 605 DAPs. Only 269 proteins were found to be DEP and DAP, of which 136 proteins were consistently expressed in both the DEP and DAP. Among them, the DEPs changed significantly in glycolysis and the citrate cycle; most of the enzymes were acetylated. The DAPs were down-regulated in glycolysis and up-regulated in the citrate cycle. Our results reveal the importance of acetylation modification in the needle senescence of *L. gmelinii* and provide a new perspective to understanding the molecular mechanisms of leaf senescence and the seasonal growth of trees.

## Data Availability

The datasets used and/or analyzed during the current study are available from the corresponding author on reasonable request.

## Supplementary Materials

The supporting information can be downloaded at: https://www.mdpi.com/article/10.3390/ijms25136824/s1.
