# Supplementary material for: Comprehensive Proteome and Acetylome Analysis of Needle Senescence in Larix gmelinii
Source: Int J Mol Sci. 2024 Jun 21;25(13):6824. doi: 10.3390/ijms25136824 (PMC11241215; doi:10.3390/ijms25136824)
Supplement: Supplementary file 1 [file ijms-25-06824-s001.zip › supplementary materials.pdf]

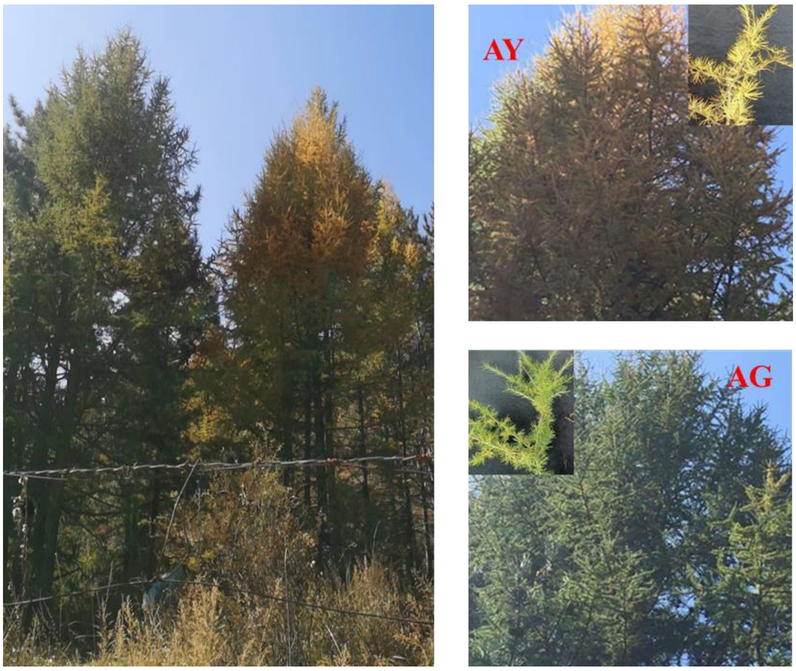

Fig. S1 The phenotype of *L. gmelinii* needle of individual tree in autumn (October 10th). Representative samples of yellow (senescing) and green (non-senescing) needles of *L. gmelinii*.

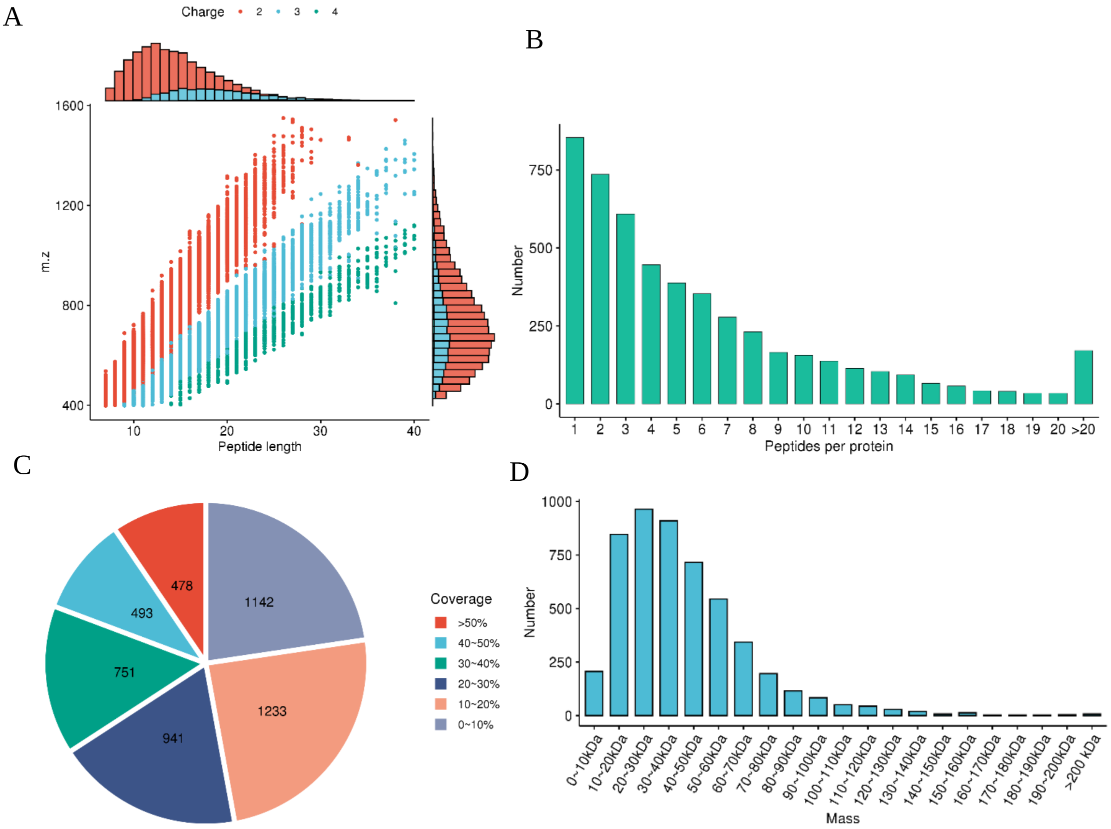

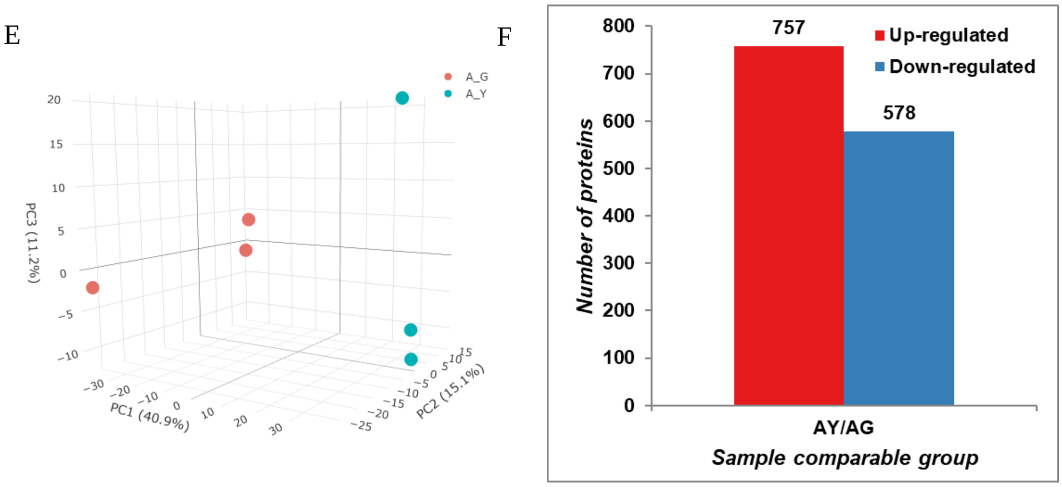

Fig. S2 Proteomic data analysis of *L. gmelinii* needle senescence. Distribution of peptide length (A) and number (B). Distribution of protein coverage (C) and molecular weight (D). Three-dimensional PCA analysis of temporal proteomic data (E). Numbers of up- and down- regulated proteins (F).

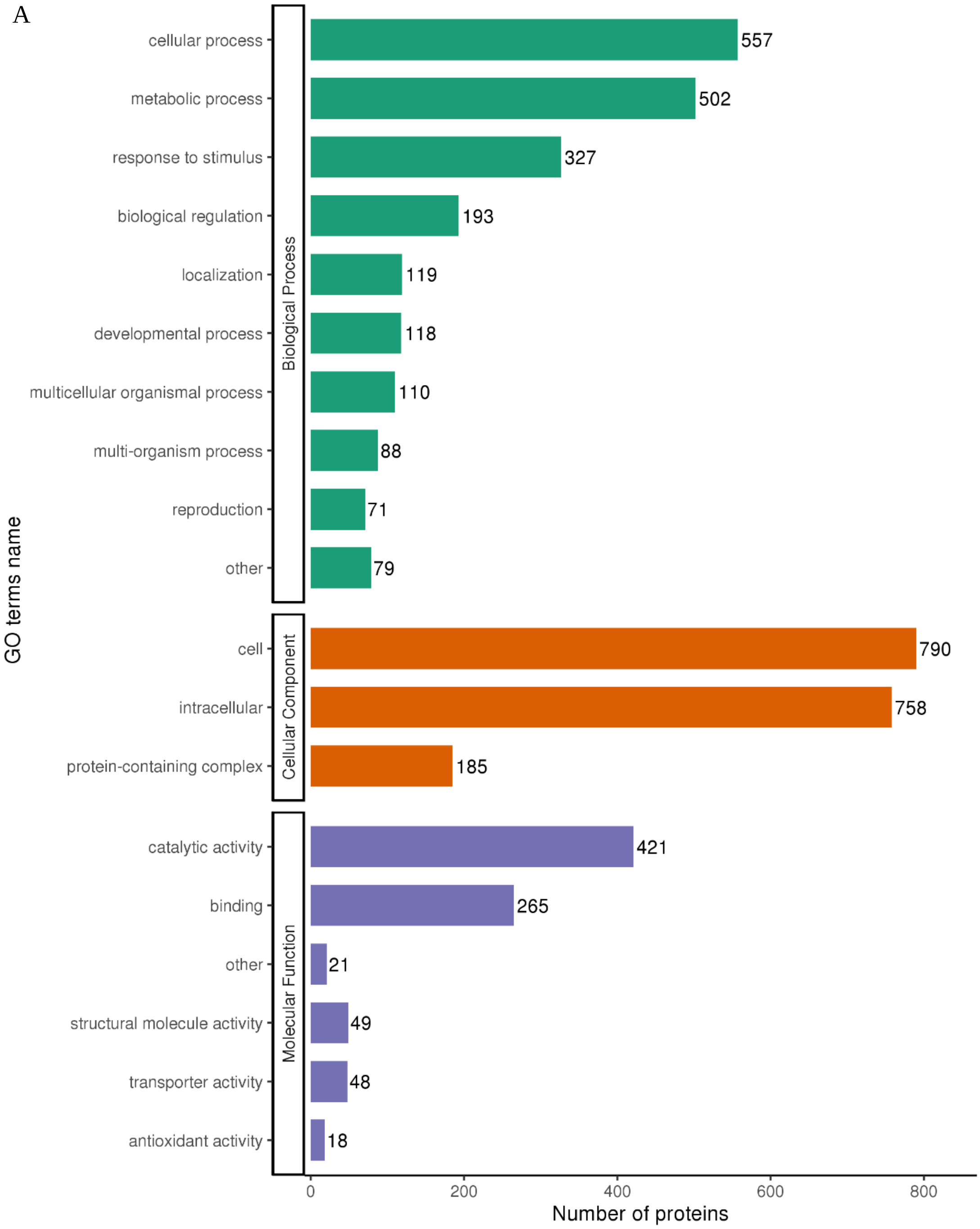
A
GO terms name
Biological Process
cellular process 557
metabolic process 502
response to stimulus 327
biological regulation 193
localization 119
developmental process 118
multicellular organismal process 110
multi-organism process 88
reproduction 71
other 79
Cellular Component
cell 790
intracellular 758
protein-containing complex 185
Molecular Function
catalytic activity 421
binding 265
other 21
structural molecule activity 49
transporter activity 48
antioxidant activity 18
0
200
400
600
800
Number of proteins

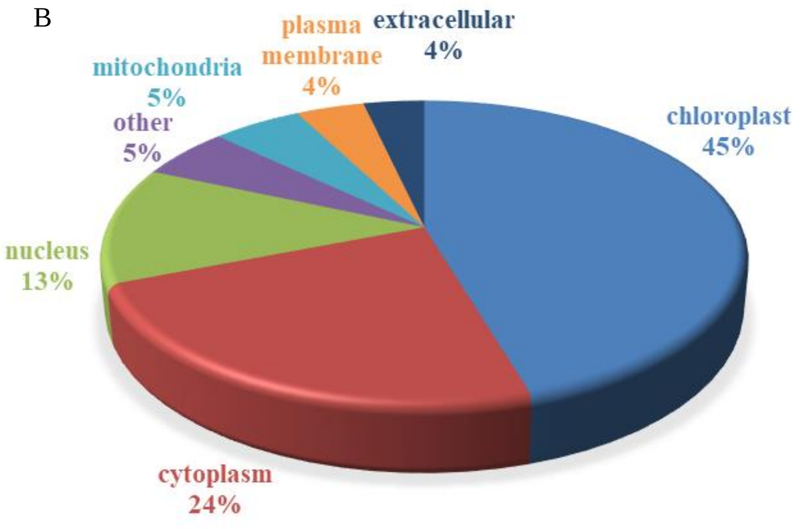

Fig. S3 Functional classification of DEPs. Gene ontology (GO) analyses of proteins by Biological process, cellular component, molecular function (A), Subcellular location(B).

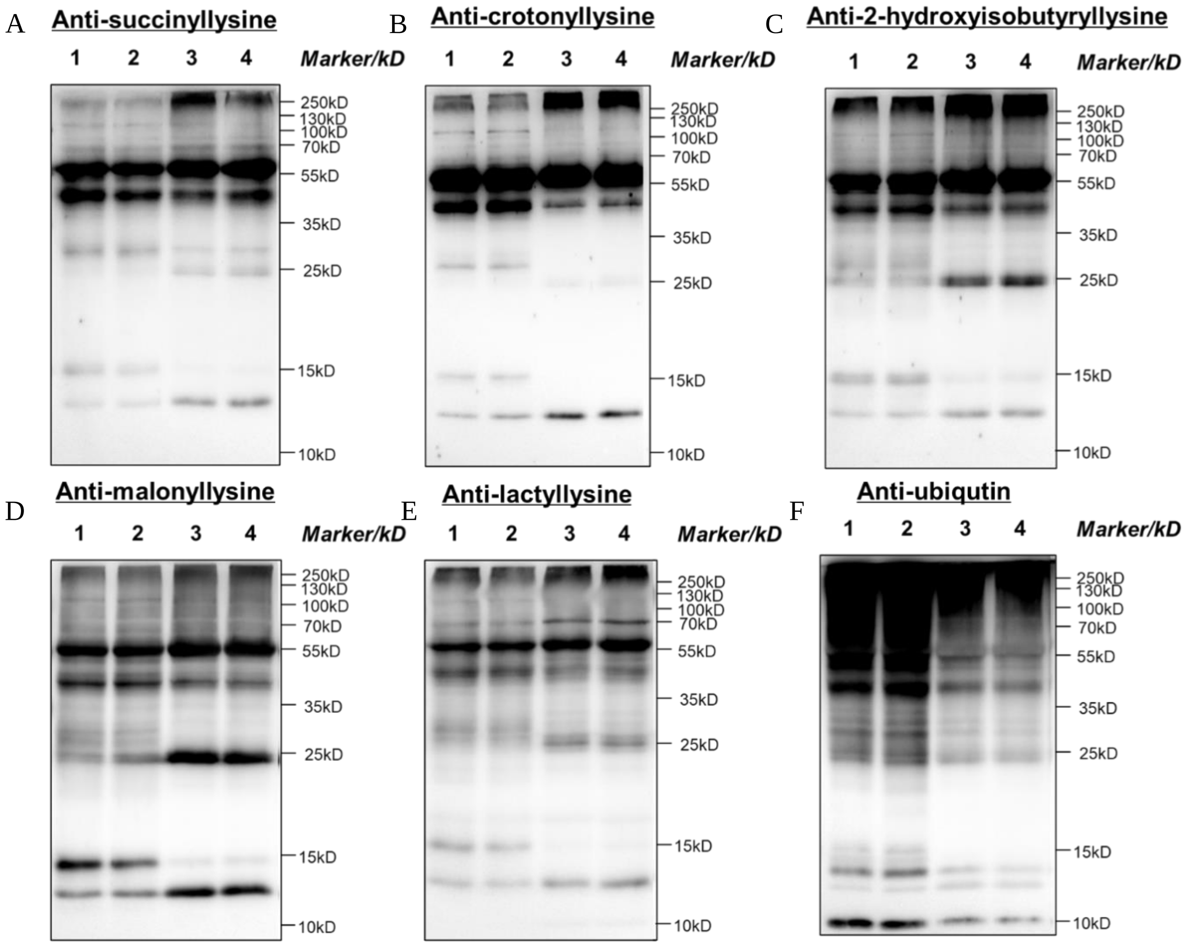

Fig. S4 Western blotting analysis of different modifications in needle senescence of *L. gmelinii*. Western blotting of the needles proteins with pan anti-succinyllysine (A), pan anti-crotonyllysine (B), pan anti-2-hydroxyisobutyryllysine (C), pan anti-malonyllysine (D), anti-lactyllysine (E), pan anti-ubiqutin (F), 1 and 2 represent two replicates of AY; 3 and 4 represent two replicates of AG.
